# Dicyclo­hexyl­ammonium 2-methoxy­benzoate

**DOI:** 10.1107/S1600536808007587

**Published:** 2008-04-02

**Authors:** Nenad Judaš, Tomislav Portada

**Affiliations:** a Department of Chemistry, Laboratory of General and Inorganic Chemistry, Faculty of Science, University of Zagreb, Horvatovac 102a, HR-10000 Zagreb, Croatia; b Department of Organic Chemistry and Biochemistry, Ruđer Bošković Institute, PO Box 180, HR-10002 Zagreb, Croatia

## Abstract

The asymmetric unit of the title compound, C_12_H_24_N^+^·C_8_H_7_O_3_^−^, contains one dicyclo­hexyl­ammonium cation and one 2-methoxy­benzoate anion. Two cations and two anions are linked together to form a four-ion cluster through a set of N—H⋯O hydrogen bonds. Weak C—H⋯O hydrogen bonds connect the clusters into chains that are stacked along the crystallographic *c* axis.

## Related literature

For the crystal structures of dicyclo­hexyl­ammonium salts of monocarboxylic acids, see: Ng *et al.* (1999 ▶); Ng, Naumov *et al.* 2001 ▶), Ng & Hook (1999 ▶); Subramanian *et al.* (2000 ▶). For the crystal structures of dicyclo­hexyl­ammonium salts of dicarboxylic acids, see: Ballabh *et al.* (2005 ▶); Trivedi *et al.* (2005 ▶); Ng, Chantrapromma *et al.* (2001 ▶). For related literature, see: Zain & Ng (2007 ▶); Trivedi *et al.* (2004 ▶); Ng *et al.* (1991 ▶); Allen *et al.* (1987 ▶).
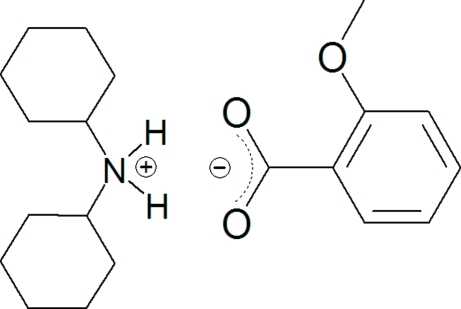


## Experimental

### 

#### Crystal data

C_12_H_24_N^+^·C_8_H_7_O_3_^−^
*M* *_r_* = 333.46
Monoclinic, 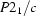

*a* = 9.2798 (5) Å
*b* = 17.7978 (9) Å
*c* = 12.1513 (7) Å
β = 104.720 (5)°
*V* = 1941.04 (18) Å^3^
*Z* = 4
Mo *K*α radiation
μ = 0.08 mm^−1^
*T* = 293 (1) K
0.62 × 0.41 × 0.35 mm

#### Data collection

Oxford Diffraction Xcalibur CCD diffractometer
Absorption correction: none
19673 measured reflections
3789 independent reflections
2750 reflections with *I* > 2σ(*I*)
*R*_int_ = 0.042

#### Refinement

*R*[*F* ^2^ > 2σ(*F* ^2^)] = 0.068
*wR*(*F* ^2^) = 0.175
*S* = 1.03
3789 reflections
225 parameters
H atoms treated by a mixture of independent and constrained refinement
Δρ_max_ = 0.37 e Å^−3^
Δρ_min_ = −0.17 e Å^−3^

### 

Data collection: *CrysAlis CCD* (Oxford Diffraction, 2003 ▶); cell refinement: *CrysAlis RED* (Oxford Diffraction, 2003 ▶); data reduction: *CrysAlis RED*; program(s) used to solve structure: *SHELXS97* (Sheldrick, 2008 ▶); program(s) used to refine structure: *SHELXL97* (Sheldrick, 2008 ▶); molecular graphics: *ORTEP-3 for Windows* (Farrugia, 1997 ▶), *Mercury* (Macrae *et al.*, 2006 ▶), *RasTop* (Valadon, 2000–2003 ▶) and *POV-RAY* (Persistence of Vision, 2004 ▶); software used to prepare material for publication: *WinGX* (Farrugia, 1999 ▶).

## Supplementary Material

Crystal structure: contains datablocks I, global. DOI: 10.1107/S1600536808007587/wn2244sup1.cif

Structure factors: contains datablocks I. DOI: 10.1107/S1600536808007587/wn2244Isup2.hkl

Additional supplementary materials:  crystallographic information; 3D view; checkCIF report

## Figures and Tables

**Table 1:** Hydrogen-bond geometry (Å, °)

*D*—H⋯*A*	*D*—H	H⋯*A*	*D*⋯*A*	*D*—H⋯*A*
N1—H2⋯O1	0.92 (3)	1.84 (3)	2.735 (3)	163 (2)
N1—H1⋯O2^i^	0.88 (2)	1.85 (3)	2.703 (2)	162 (2)
C20—H20*A*⋯O1^ii^	0.97	2.66	3.457 (3)	140

Symmetry codes: (i) 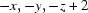; (ii) 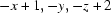.

**Fig. 1.**
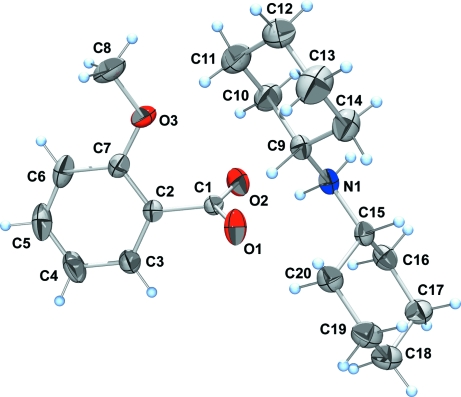
The molecular structure of the title compound, showing the atomic numbering scheme. Displacement ellipsoids are drawn at the 30% probability level and H atoms are shown as small spheres of arbitrary radius.

**Fig. 2.**
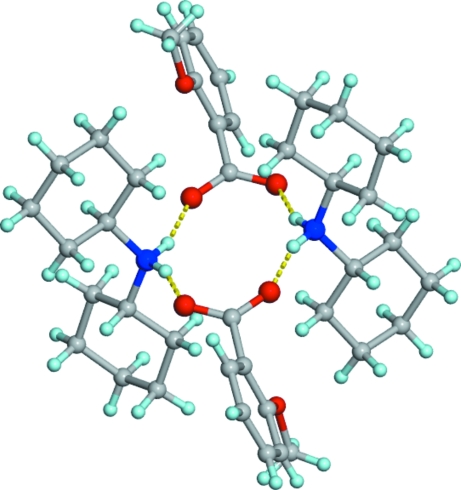
Self-assembly of cations and anions through N1—H2···O1 and N1—H1···O2 hydrogen bonds into tetrameric units.

**Fig. 3.**
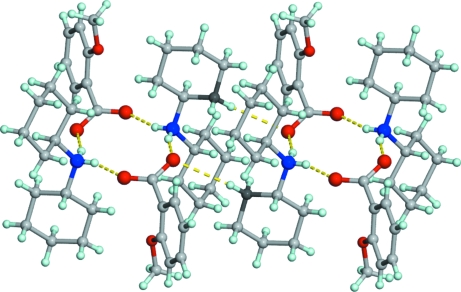
Linkage of the tetrameric units into molecular chains through weak C20—H20···O1 hydrogen bonds. Carbon atoms C20 involved in hydrogen bonding are darkened for clarity.

**Fig. 4.**
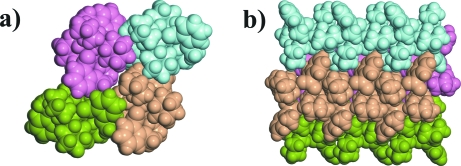
Views of the crystal structure of the title compound depicting: (a) the narrow channels between neighboring chains of tetrameric units; (b) the interpenetration of benzene rings belonging to neighboring chains. Atoms of each chain have been color-coded for clarity.

## supplementary crystallographic information

### Comment

The title compound was synthesized as a model for the purposes of a workshop on parallel synthesis and combinatorial chemistry. The compound was selected because of its resemblance to dicyclohexylammonium salts of substituted cinnamic acids, that are widely known as gelators of organic fluids (Ballabh *et al.*, 2005; Trivedi *et al.*, 2005, Trivedi *et al.*, 2004).

The molecular structure of the title compound is shown in Fig. 1. The asymmetric unit consist of a dicyclohexylammonium cation and a 2-methoxybenzoate anion. The carboxylate group of the anion is twisted with respect to the parent benzene ring by 65.1 (2)°. All bond lengths fall within normal ranges (Allen *et al.*, 1987).

Two cations and two anions self-assemble into a tetrameric structural unit by two hydrogen bonds; N1—H2···O1 and N1—H1···O2*^i^* (Fig. 2, Table 1.).

Weak C20—H20···O1*^ii^* hydrogen bonds (Fig. 3, Table 1) link these tetrameric units into chains that are stacked together in a zipper-like manner, so as to produce narrow channels between them (Fig. 4a). The appearance of the channels is consistent with the relatively low calculated density of the title compound (1.14 g cm^-3^).

The zipper-like stacking is achieved by the interdigitation of protruding benzene groups in each chain (Fig. 4 b), thus maximizing the intermolecular contacts.

### Experimental

A solution of dicyclohexylamine (363 mg, 2.00 mmol) in toluene (5 ml) was added with stirring to a solution of 2-methoxybenzoic acid (304 mg, 2.00 mmol) in toluene (5 ml). The resulting solution was allowed to stand in an open beaker for several days until crystals of the title compound formed by slow solvent evaporation. The crystals were suitable for single-crystal X-ray diffraction. The compound was also analyzed by thermal methods (TG and DSC). Thermal analyses were performed on METTLER thermal analysis modules DSC823*^e^* and TGA/SDTA851*^e^*. The calorimetric thermogram exhibited one endothermic signal that was sharp and well defined, corresponding to the melting point of the compound. The onset temperature of the signal is *T*_f_ = 416 K with enthalpy of fusion, Δ*H*_fus_ = 37,9 kJ mol^-1^. Degradation of the sample begins above 524 K.

### Refinement

Carbon-bound H atoms were placed in calculated positions and included in the refinement using the riding-model approximation, with C—H distances of 0.93 Å for phenyl, 0.97 Å for methylene, 0.98 Å for methine and 0.96 Å for methyl groups, and with *U*_iso_(H) = 1.2*U*_eq_(C) or 1.2*U*_eq_(C_methyl_). A rotating group model was used for the methyl groups. The hydrogen atoms of the amine group were located in the final Fourier difference map and their coordinates were blocked during the refinement process.

### Crystal data

**Table:**

C_12_H_24_N^+^·C_8_H_7_O_3_^–^	*D*_x_ = 1.141 Mg m^−^^3^
*M**_r_* = 333.46	Melting point: 416 K
Monoclinic, *P*2_1_/*c*	Mo *K*α radiation λ = 0.71073 Å
Hall symbol: -P 2ybc	Cell parameters from 742 reflections
*a* = 9.2798 (5) Å	θ = 6.4–21.2º
*b* = 17.7978 (9) Å	µ = 0.08 mm^−^^1^
*c* = 12.1513 (7) Å	*T* = 293 (1) K
β = 104.720 (5)º	Cell measurement pressure: 101(1) kPa
*V* = 1941.04 (18) Å^3^	Prismatic, colourless
*Z* = 4	0.62 × 0.41 × 0.35 mm
*F*_000_ = 728	

### Data collection

**Table:**

Oxford Diffraction Xcalibur CCD diffractometer	3789 independent reflections
Radiation source: fine-focus sealed tube	2750 reflections with *I* > 2σ(*I*)
Monochromator: graphite	*R*_int_ = 0.042
*T* = 293(1) K	θ_max_ = 26.0º
*P* = 101(1) kPa	θ_min_ = 4.1º
ω scans	*h* = −11→11
Absorption correction: none	*k* = −21→21
19673 measured reflections	*l* = −14→14

### Refinement

**Table:**

Refinement on *F*^2^	Hydrogen site location: inferred from neighbouring sites
Least-squares matrix: full	H atoms treated by a mixture of independent and constrained refinement
*R*[*F*^2^ > 2σ(*F*^2^)] = 0.068	*w* = 1/[σ^2^(*F*_o_^2^) + (0.0745*P*)^2^ + 0.785*P*] where *P* = (*F*_o_^2^ + 2*F*_c_^2^)/3
*wR*(*F*^2^) = 0.175	(Δ/σ)_max_ < 0.001
*S* = 1.03	Δρ_max_ = 0.37 e Å^−^^3^
3789 reflections	Δρ_min_ = −0.16 e Å^−^^3^
225 parameters	Extinction correction: SHELXL, Fc^*^=kFc[1+0.001xFc^2^λ^3^/sin(2θ)]^-1/4^
Primary atom site location: structure-invariant direct methods	Extinction coefficient: 0.034 (4)
Secondary atom site location: difference Fourier map	

### Special details

**Table:**

Geometry. All e.s.d.'s (except the e.s.d. in the dihedral angle between two l.s. planes) are estimated using the full covariance matrix. The cell e.s.d.'s are taken into account individually in the estimation of e.s.d.'s in distances, angles and torsion angles; correlations between e.s.d.'s in cell parameters are only used when they are defined by crystal symmetry. An approximate (isotropic) treatment of cell e.s.d.'s is used for estimating e.s.d.'s involving l.s. planes.
Refinement. Refinement of *F*^2^ against ALL reflections. The weighted *R*-factor *wR* and goodness of fit *S* are based on *F*^2^, conventional *R*-factors *R* are based on *F*, with *F* set to zero for negative *F*^2^. The threshold expression of *F*^2^ > σ(*F*^2^) is used only for calculating *R*-factors(gt) *etc*. and is not relevant to the choice of reflections for refinement. *R*-factors based on *F*^2^ are statistically about twice as large as those based on *F*, and *R*- factors based on ALL data will be even larger.

### Fractional atomic coordinates and isotropic or equivalent isotropic displacement parameters (Å^2^)

**Table:**

	*x*	*y*	*z*	*U*_iso_*/*U*_eq_	
O1	0.2706 (2)	0.08567 (10)	1.00183 (17)	0.0842 (6)	
O2	0.04537 (19)	0.13445 (12)	0.96352 (17)	0.0852 (6)	
O3	0.0699 (2)	0.14787 (12)	0.72365 (14)	0.0819 (6)	
N1	0.2059 (2)	−0.06289 (11)	1.02163 (16)	0.0491 (5)	
H1	0.114 (3)	−0.0780 (13)	1.0175 (19)	0.059*	
H2	0.209 (2)	−0.0112 (15)	1.0178 (19)	0.059*	
C1	0.1749 (2)	0.13270 (11)	0.95593 (17)	0.0471 (5)	
C2	0.2251 (2)	0.19520 (11)	0.89133 (17)	0.0438 (5)	
C3	0.3308 (3)	0.24641 (13)	0.9464 (2)	0.0634 (6)	
H3	0.3731	0.2413	1.0241	0.076*	
C4	0.3748 (4)	0.30529 (15)	0.8877 (3)	0.0898 (10)	
H4	0.4465	0.3392	0.9258	0.108*	
C5	0.3122 (4)	0.31337 (16)	0.7736 (3)	0.0894 (10)	
H5	0.3400	0.3535	0.7345	0.107*	
C6	0.2088 (3)	0.26265 (16)	0.7164 (2)	0.0750 (8)	
H6	0.1671	0.2682	0.6388	0.090*	
C7	0.1666 (2)	0.20300 (13)	0.77480 (19)	0.0537 (6)	
C8	0.0317 (4)	0.1435 (2)	0.6036 (2)	0.0992 (11)	
H8B	−0.0221	0.1878	0.5722	0.149*	
H8C	−0.0295	0.1000	0.5795	0.149*	
H8A	0.1208	0.1395	0.5776	0.149*	
C9	0.2458 (2)	−0.09394 (12)	0.91808 (18)	0.0507 (5)	
H9	0.3437	−0.0742	0.9162	0.061*	
C10	0.1326 (3)	−0.06616 (16)	0.8135 (2)	0.0761 (7)	
H10A	0.1324	−0.0117	0.8129	0.091*	
H10B	0.0340	−0.0830	0.8159	0.091*	
C11	0.1681 (4)	−0.09527 (19)	0.7053 (2)	0.0940 (10)	
H11B	0.0913	−0.0787	0.6397	0.113*	
H11A	0.2622	−0.0742	0.6992	0.113*	
C12	0.1775 (4)	−0.17925 (19)	0.7044 (3)	0.0950 (10)	
H12B	0.2082	−0.1956	0.6377	0.114*	
H12A	0.0800	−0.2005	0.7005	0.114*	
C13	0.2877 (5)	−0.2071 (2)	0.8103 (3)	0.1075 (11)	
H13A	0.3869	−0.1911	0.8086	0.129*	
H13B	0.2865	−0.2616	0.8106	0.129*	
C14	0.2540 (3)	−0.17841 (14)	0.9200 (2)	0.0760 (8)	
H14B	0.1600	−0.1991	0.9271	0.091*	
H14A	0.3316	−0.1948	0.9852	0.091*	
C15	0.3019 (2)	−0.08294 (11)	1.13645 (17)	0.0471 (5)	
H15	0.2968	−0.1374	1.1467	0.057*	
C16	0.2390 (2)	−0.04421 (14)	1.2256 (2)	0.0599 (6)	
H16A	0.2334	0.0094	1.2110	0.072*	
H16B	0.1386	−0.0623	1.2194	0.072*	
C17	0.3332 (3)	−0.05828 (17)	1.3448 (2)	0.0743 (7)	
H17B	0.3262	−0.1109	1.3637	0.089*	
H17A	0.2947	−0.0287	1.3981	0.089*	
C18	0.4956 (3)	−0.03803 (17)	1.3571 (2)	0.0755 (7)	
H18A	0.5047	0.0159	1.3490	0.091*	
H18B	0.5541	−0.0522	1.4323	0.091*	
C19	0.5553 (3)	−0.07794 (15)	1.2679 (2)	0.0717 (7)	
H19B	0.6573	−0.0623	1.2748	0.086*	
H19A	0.5555	−0.1317	1.2812	0.086*	
C20	0.4629 (2)	−0.06121 (14)	1.1488 (2)	0.0594 (6)	
H20A	0.5021	−0.0890	1.0941	0.071*	
H20B	0.4689	−0.0080	1.1329	0.071*	

### Atomic displacement parameters (Å^2^)

**Table:**

	*U*^11^	*U*^22^	*U*^33^	*U*^12^	*U*^13^	*U*^23^
O1	0.1052 (14)	0.0553 (11)	0.1037 (15)	0.0129 (10)	0.0479 (12)	0.0276 (10)
O2	0.0615 (10)	0.1125 (16)	0.0879 (14)	−0.0220 (10)	0.0307 (9)	0.0165 (11)
O3	0.0777 (11)	0.1234 (17)	0.0406 (9)	−0.0256 (11)	0.0079 (8)	−0.0009 (9)
N1	0.0455 (9)	0.0437 (10)	0.0621 (12)	0.0020 (8)	0.0210 (8)	0.0081 (8)
C1	0.0600 (12)	0.0460 (12)	0.0374 (11)	−0.0104 (10)	0.0166 (9)	−0.0060 (9)
C2	0.0455 (10)	0.0423 (11)	0.0465 (12)	0.0037 (8)	0.0168 (9)	0.0004 (8)
C3	0.0789 (16)	0.0585 (14)	0.0571 (14)	−0.0167 (12)	0.0252 (12)	−0.0057 (11)
C4	0.121 (2)	0.0557 (16)	0.108 (3)	−0.0344 (16)	0.056 (2)	−0.0122 (15)
C5	0.127 (3)	0.0545 (16)	0.108 (3)	0.0052 (17)	0.068 (2)	0.0244 (16)
C6	0.0877 (18)	0.0762 (18)	0.0692 (17)	0.0236 (15)	0.0344 (14)	0.0327 (14)
C7	0.0502 (11)	0.0625 (14)	0.0503 (13)	0.0092 (10)	0.0164 (10)	0.0082 (10)
C8	0.091 (2)	0.152 (3)	0.0475 (16)	0.004 (2)	0.0051 (14)	−0.0046 (18)
C9	0.0505 (11)	0.0522 (12)	0.0529 (13)	−0.0041 (9)	0.0196 (10)	0.0033 (9)
C10	0.0922 (19)	0.0692 (17)	0.0626 (17)	0.0069 (14)	0.0116 (14)	0.0055 (13)
C11	0.125 (3)	0.091 (2)	0.0612 (18)	−0.0079 (19)	0.0165 (17)	0.0017 (15)
C12	0.121 (3)	0.093 (2)	0.071 (2)	−0.0183 (19)	0.0259 (18)	−0.0164 (17)
C13	0.164 (3)	0.078 (2)	0.088 (2)	0.027 (2)	0.046 (2)	−0.0125 (17)
C14	0.107 (2)	0.0544 (15)	0.0715 (18)	0.0130 (14)	0.0313 (15)	0.0015 (12)
C15	0.0506 (11)	0.0382 (10)	0.0542 (13)	0.0017 (8)	0.0165 (10)	0.0060 (9)
C16	0.0547 (12)	0.0597 (14)	0.0712 (16)	−0.0047 (10)	0.0268 (11)	−0.0068 (11)
C17	0.0851 (18)	0.0795 (18)	0.0591 (16)	−0.0140 (14)	0.0200 (13)	−0.0135 (13)
C18	0.0735 (16)	0.0733 (17)	0.0737 (18)	−0.0050 (13)	0.0074 (13)	−0.0142 (14)
C19	0.0571 (13)	0.0656 (16)	0.0864 (19)	0.0069 (12)	0.0071 (13)	−0.0050 (14)
C20	0.0476 (11)	0.0629 (14)	0.0698 (16)	0.0050 (10)	0.0189 (11)	0.0007 (11)

### Geometric parameters (Å, °)

**Table:**

O1—C1	1.244 (3)	C11—C12	1.498 (5)
O2—C1	1.229 (3)	C11—H11B	0.9700
O3—C7	1.368 (3)	C11—H11A	0.9700
O3—C8	1.413 (3)	C12—C13	1.510 (5)
N1—C15	1.495 (3)	C12—H12B	0.9700
N1—C9	1.504 (3)	C12—H12A	0.9700
N1—H1	0.88 (2)	C13—C14	1.532 (4)
N1—H2	0.92 (3)	C13—H13A	0.9700
C1—C2	1.502 (3)	C13—H13B	0.9700
C2—C3	1.381 (3)	C14—H14B	0.9700
C2—C7	1.389 (3)	C14—H14A	0.9700
C3—C4	1.386 (4)	C15—C20	1.513 (3)
C3—H3	0.9300	C15—C16	1.520 (3)
C4—C5	1.368 (4)	C15—H15	0.9800
C4—H4	0.9300	C16—C17	1.511 (3)
C5—C6	1.371 (4)	C16—H16A	0.9700
C5—H5	0.9300	C16—H16B	0.9700
C6—C7	1.387 (3)	C17—C18	1.520 (4)
C6—H6	0.9300	C17—H17B	0.9700
C8—H8B	0.9600	C17—H17A	0.9700
C8—H8C	0.9600	C18—C19	1.513 (4)
C8—H8A	0.9600	C18—H18A	0.9700
C9—C14	1.505 (3)	C18—H18B	0.9700
C9—C10	1.511 (3)	C19—C20	1.513 (3)
C9—H9	0.9800	C19—H19B	0.9700
C10—C11	1.526 (4)	C19—H19A	0.9700
C10—H10A	0.9700	C20—H20A	0.9700
C10—H10B	0.9700	C20—H20B	0.9700
			
C7—O3—C8	118.2 (2)	C11—C12—H12B	109.5
C15—N1—C9	118.55 (16)	C13—C12—H12B	109.5
C15—N1—H1	108.5 (15)	C11—C12—H12A	109.5
C9—N1—H1	106.2 (15)	C13—C12—H12A	109.5
C15—N1—H2	105.4 (14)	H12B—C12—H12A	108.1
C9—N1—H2	107.8 (14)	C12—C13—C14	112.8 (3)
H1—N1—H2	110 (2)	C12—C13—H13A	109.0
O2—C1—O1	125.7 (2)	C14—C13—H13A	109.0
O2—C1—C2	117.3 (2)	C12—C13—H13B	109.0
O1—C1—C2	116.86 (18)	C14—C13—H13B	109.0
C3—C2—C7	118.3 (2)	H13A—C13—H13B	107.8
C3—C2—C1	120.77 (19)	C9—C14—C13	109.9 (2)
C7—C2—C1	120.94 (18)	C9—C14—H14B	109.7
C2—C3—C4	121.0 (3)	C13—C14—H14B	109.7
C2—C3—H3	119.5	C9—C14—H14A	109.7
C4—C3—H3	119.5	C13—C14—H14A	109.7
C5—C4—C3	119.8 (3)	H14B—C14—H14A	108.2
C5—C4—H4	120.1	N1—C15—C20	111.74 (17)
C3—C4—H4	120.1	N1—C15—C16	108.11 (17)
C4—C5—C6	120.4 (2)	C20—C15—C16	111.23 (18)
C4—C5—H5	119.8	N1—C15—H15	108.6
C6—C5—H5	119.8	C20—C15—H15	108.6
C5—C6—C7	119.8 (3)	C16—C15—H15	108.6
C5—C6—H6	120.1	C17—C16—C15	112.00 (19)
C7—C6—H6	120.1	C17—C16—H16A	109.2
O3—C7—C6	123.7 (2)	C15—C16—H16A	109.2
O3—C7—C2	115.66 (19)	C17—C16—H16B	109.2
C6—C7—C2	120.6 (2)	C15—C16—H16B	109.2
O3—C8—H8B	109.5	H16A—C16—H16B	107.9
O3—C8—H8C	109.5	C16—C17—C18	111.9 (2)
H8B—C8—H8C	109.5	C16—C17—H17B	109.2
O3—C8—H8A	109.5	C18—C17—H17B	109.2
H8B—C8—H8A	109.5	C16—C17—H17A	109.2
H8C—C8—H8A	109.5	C18—C17—H17A	109.2
N1—C9—C14	112.10 (18)	H17B—C17—H17A	107.9
N1—C9—C10	108.46 (18)	C19—C18—C17	110.6 (2)
C14—C9—C10	111.2 (2)	C19—C18—H18A	109.5
N1—C9—H9	108.3	C17—C18—H18A	109.5
C14—C9—H9	108.3	C19—C18—H18B	109.5
C10—C9—H9	108.3	C17—C18—H18B	109.5
C9—C10—C11	110.9 (2)	H18A—C18—H18B	108.1
C9—C10—H10A	109.5	C20—C19—C18	111.8 (2)
C11—C10—H10A	109.5	C20—C19—H19B	109.3
C9—C10—H10B	109.5	C18—C19—H19B	109.3
C11—C10—H10B	109.5	C20—C19—H19A	109.3
H10A—C10—H10B	108.0	C18—C19—H19A	109.3
C12—C11—C10	111.7 (3)	H19B—C19—H19A	107.9
C12—C11—H11B	109.3	C19—C20—C15	110.46 (19)
C10—C11—H11B	109.3	C19—C20—H20A	109.6
C12—C11—H11A	109.3	C15—C20—H20A	109.6
C10—C11—H11A	109.3	C19—C20—H20B	109.6
H11B—C11—H11A	107.9	C15—C20—H20B	109.6
C11—C12—C13	110.5 (3)	H20A—C20—H20B	108.1

### Hydrogen-bond geometry (Å, °)

**Table:**

*D*—H···*A*	*D*—H	H···*A*	*D*···*A*	*D*—H···*A*
N1—H2···O1	0.92 (3)	1.84 (3)	2.735 (3)	163 (2)
N1—H1···O2^i^	0.88 (2)	1.85 (3)	2.703 (2)	162 (2)
C20—H20A···O1^ii^	0.97	2.66	3.457 (3)	140

Symmetry codes: (i) −*x*, −*y*, −*z*+2; (ii) −*x*+1, −*y*, −*z*+2.

## References

[bb1] Allen, F. H., Kennard, O., Watson, D. G., Brammer, L., Orpen, A. G. & Taylor, R. (1987). *J. Chem. Soc. Perkin Trans. 2*, pp. S1–S19.

[bb2] Ballabh, A., Trivedi, D. R. & Dastidar, P. (2005). *Cryst. Growth Des.* **5**, 1545–1553.

[bb3] Farrugia, L. J. (1997). *J. Appl. Cryst.* **30**, 565.

[bb4] Farrugia, L. J. (1999). *J. Appl. Cryst.* **32**, 837–838.

[bb5] Macrae, C. F., Edgington, P. R., McCabe, P., Pidcock, E., Shields, G. P., Taylor, R., Towler, M. & van de Streek, J. (2006). *J. Appl. Cryst.* **39**, 453–457.

[bb6] Ng, S. W., Chantrapromma, S., Razak, I. A. & Fun, H.-K. (2001). *Acta Cryst.* C**57**, 291–292. 10.1107/s0108270100019326 11250582

[bb7] Ng, S. W., Fun, H.-K. & Shanmuga Sundara Raj, S. (1999). *Acta Cryst.* C**55**, 2145–2147.

[bb8] Ng, S. W. & Hook, J. M. (1999). *Acta Cryst.* C**55**, 312–316.

[bb9] Ng, S. W., Kumar Das, V. G. & Tiekink, E. R. T. (1991). *J. Organomet. Chem.* **411**, 121–129.

[bb10] Ng, S. W., Naumov, P., Drew, M. G. B., Wojciechowski, G. & Brzezinski, B. (2001). *J. Mol. Struct.* **595**, 29–37.

[bb11] Oxford Diffraction (2003). *CrysAlis CCD* and *CrysAlis RED* Oxford Diffraction Ltd, Wroclaw, Poland.

[bb12] Persistence of Vision (2004). *POV-RAY.* Persistence of Vision Raytracer Pty Ltd, Victoria, Australia.

[bb13] Sheldrick, G. M. (2008). *Acta Cryst.* A**64**, 112–122. 10.1107/S0108767307043930 18156677

[bb14] Subramanian, R. R., Anandan, S. S., Kwek, K. H., Low, K. S., Shanmuga Sundara Raj, S., Fun, H.-K., Razak, I. A., Hanna, J. V. & Ng, S. W. (2000). *Acta Cryst.* C**56**, e292–e294.

[bb15] Trivedi, D. R., Ballabh, A. & Dastidar, P. (2004). *Chem. Eur. J.* **10**, 5311–5322. 10.1002/chem.200400122 15378683

[bb16] Trivedi, D. R., Ballabh, A. & Dastidar, P. (2005). *J. Mater. Chem.* **5**, 1545–1553.

[bb17] Valadon, P. (2000–2003). *RasTop* Phillipe Valadon, **Location?**

[bb18] Zain, S. M. & Ng, S. W. (2007). *Acta Cryst.* E**63**, o3303.

